# A Solid-Phase Microextraction—Liquid Chromatography-Mass Spectrometry Method for Analyzing Serum Lipids in Psoriatic Disease

**DOI:** 10.3390/metabo13080963

**Published:** 2023-08-20

**Authors:** John Koussiouris, Nikita Looby, Vathany Kulasingam, Vinod Chandran

**Affiliations:** 1 Schroeder Arthritis Institute, Krembil Research Institute, University Health Network, Toronto, ON M5T 0S8, Canada; john.koussiouris@uhnresearch.ca (J.K.); nikita.looby@uhnresearch.ca (N.L.); 2 Department of Laboratory Medicine and Pathobiology, University of Toronto, Toronto, ON M5S 1A8, Canada; vathany.kulasingam@uhn.ca; 3 Division of Clinical Biochemistry, Laboratory Medicine Program, University Health Network, Toronto, ON M5G 2C4, Canada; 4 Division of Rheumatology, Department of Medicine, University of Toronto, Toronto, ON M5S 1A8, Canada; 5 Institute of Medical Science, University of Toronto, Toronto, ON M5S 1A8, Canada; 6 Department of Medicine, Memorial University, St. John’s, NL A1B 3V6, Canada

**Keywords:** psoriatic disease, metabolomics, lipids, solid-phase microextraction, liquid chromatography, mass spectrometry

## Abstract

Approximately 25% of psoriasis patients have an inflammatory arthritis termed psoriatic arthritis (PsA). There is strong interest in identifying and validating biomarkers that can accurately and reliably predict conversion from psoriasis to PsA using novel technologies such as metabolomics. Lipids, in particular, are of key interest in psoriatic disease. We sought to develop a liquid chromatography-mass spectrometry (LC-MS) method to be used in conjunction with solid-phase microextraction (SPME) for analyzing fatty acids and similar molecules. A total of 25 chromatographic methods based on published lipid studies were tested on two LC columns. As a proof of concept, serum samples from psoriatic disease patients (n = 27 psoriasis and n = 26 PsA) were processed using SPME and run on the selected LC-MS method. The method that was best for analyzing fatty acids and fatty acid-like molecules was optimized and applied to serum samples. A total of 18 tentatively annotated features classified as fatty acids and other lipid compounds were statistically significant between psoriasis and PsA groups using both multivariate and univariate approaches. The SPME-LC-MS method developed and optimized was capable of detecting fatty acids and similar lipids that may aid in differentiating psoriasis and PsA patients.

## 1. Introduction

Psoriasis is an inflammatory skin disease that affects approximately 10 million Americans and 1 million Canadians [1]. The most common form of psoriasis is chronic plaque psoriasis, which clinically manifests as well-demarcated erythematous scaly plaques affecting the skin. Approximately a quarter of psoriasis patients have an inflammatory arthritis termed psoriatic arthritis (PsA) [2], which is linked to reduced quality of life and limited functional capacity [3]. PsA presents clinically with peripheral joint involvement, enthesitis, tendonitis, dactylitis, inflammatory spinal disease, and other extra-musculoskeletal features [4]. Psoriatic disease patients often present with comorbidities such as cardiovascular disease, obesity, and hyperlipidemia. These conditions are more prevalent in PsA patients than in patients with psoriasis without PsA (PsC) [5].

Recently, there has been growing interest in exploring predictive biological markers (biomarkers) for psoriatic disease, with a specific focus on PsA [6]. Biomarker discovery may help elucidate unexplored mechanisms behind psoriatic disease pathogenesis and may help identify novel disease targets. Biomarker research in PsA is in the beginning stages of exploration and has focused mostly on single modality ‘omic’ studies [7]. There have been a few markers discovered that have been shown to be associated with PsA diagnosis via untargeted genomic, transcriptomic, and proteomic studies; however, the predictive value of these markers has not been sufficient to achieve the high threshold required for a diagnostic test [7]. Future biomarker research in psoriatic disease is transitioning in the direction of additional technologies such as metabolomics.

Metabolomics is an emerging field of ‘omics’ sciences that systematically investigates the diverse array of small molecules (<1500 Da) present in a biological system, including but not limited to sugars, nucleotides, amino acids, organic and inorganic acids, xenobiotics, and lipids. The metabolome is a rapid indicator of biological status [8]. In contrast to the genome and transcriptome, where genetic changes take longer to take effect, the metabolome is dynamic and constantly changing. Thus, the metabolome provides a snapshot of an organism’s physiological status at a specific point in time. Furthermore, the metabolome reflects not only the genome but also the environment and microbiome [9]. Metabolomics provides the opportunity to observe interactions between all these factors, which may play a role in the disease’s pathophysiology. Therefore, the metabolome can be regarded as the best reflection of the organism’s observable phenotype. Metabolomics in combination with other ‘omics’ scientific studies can be a powerful strategy for capturing the genetic, immunologic, and environmental factors that lead to PsA pathogenesis and may aid in biomarker discovery.

A scoping review of the literature revealed that the vast majority of metabolomic studies published in psoriatic disease have identified amino acids and lipids that may be associated with psoriasis diagnosis and activity [6]. Very few studies have examined the metabolome of PsA patients; however, the few that have have reported keto acid and fatty acid dysregulation in PsA compared to PsC [6]. Furthermore, a previous preliminary untargeted metabolomics study revealed that serum levels of select lipids, indicative of dysregulation of fatty acid metabolism, may be associated with PsA activity [10]. As such, there is a definite need to identify and further validate biomarkers that can accurately and reliably predict disease diagnosis, disease activity, and disease conversion from PsC to PsA [6]. Thus, a new untargeted metabolomics approach geared towards the separation of fatty acids from PsA patients is needed.

In order to develop and undergo an untargeted metabolomic study, an appropriate workflow must be selected [6]. The most common analytical instruments used to perform metabolomics are nuclear magnetic resonance (NMR) and mass spectrometry (MS), paired with either gas chromatography (GC-MS) or liquid chromatography (LC-MS) for separation prior to MS detection. Over time, however, MS-based platforms have become the preferred analytical technique in metabolomics due to their high sensitivity, comprehensive metabolite coverage, smaller sample volume requirements, and lower initial expense in comparison to NMR [6,11].

LC separates analytes in a sample based on their interactions with the mobile and stationary phases, while MS measures the molecular weight of an analyte in relation to its charge (mass-to-charge [*m*/*z*] ratio). The capability of LC-MS to detect most semi- and non-volatile organic molecules from a range of biological fluids and tissues has rendered it the most utilized technique for capturing lipid metabolites [6,12]. For example, Chen et al. utilized LC-MS to identify a panel of serum phosphatidylcholines and phosphatidylethanolamines capable of differentiating patients with early stage non-small cell lung cancer from healthy controls [13]. Similarly, Zhang et al. employed a targeted LC-MS/MS metabolomics approach to identify several plasma fatty acid metabolites as candidate markers of Parkinson’s disease [14].

While negligible sample preparation is required for NMR-based metabolomics, sample preparation is especially important for GC-MS and LC-MS metabolomic platforms, as these techniques necessitate substantial sample clean-up to prevent clogging, fouling, or potential damage to the instruments [15,16]. The aim of sample preparation in metabolomics is to establish an appropriate quenching mechanism and isolate metabolites of interest effectively and exclusively from complex samples [15,16]. Traditional sample preparation techniques have included liquid-liquid extraction (LLE) and solid-phase extraction (SPE). Compounds are separated in LLE based on their solubilities in two immiscible liquids, while SPE employs a cartridge with a solid adsorbent to extract analytes from a sample [15,17]. LLE is simple, fast, and does not require specialized equipment; however, the method requires a large solvent-to-sample ratio, uses environmentally toxic organic solvents, and is not easily automated [6]. SPE, on the other hand, consumes fewer environmentally toxic solvents, offers superior sample clean-up, and facilitates easier automation; however, the selection of a solid sorbent increases selectivity for specific metabolites, ultimately reducing the coverage of metabolite classes [6].

Unlike conventional sample preparation methods, solid-phase microextraction (SPME) is a novel, non-exhaustive technique that extracts only a small portion of free analytes from a sample [18]. A solid support (fiber) with a polymer coating is exposed to a sample for a preset amount of time, during which analytes migrate from the sample matrix onto the coating [19]. An equilibrium is then established between the fiber coating and sample matrix, whereby the amount of the analyte in/on the coating is proportional to the concentration of that analyte in the sample matrix [19]. Multiple forms of functionalized polymer particles with varying extraction capabilities exist for SPME [20]. After an extensive evaluation of multiple SPME coatings, Liu et al. discovered that a combination of PS-DVB and HLB coatings extracted the largest range of analytes, including acidic, basic, and neutral compounds with a wide spectrum of log P values [20]. As such, SPME is a promising sample preparation method to perform prior to LC-MS untargeted metabolomic studies given its capabilities for non-selective extraction of a wide range of hydrophilic and hydrophobic metabolites from multiple forms of complex matrices (liquid, gaseous, and solid samples) [15,19]. This rapid technique is less labor intensive, has a more streamlined workflow, and is amenable to minimal lab footprint automation and semi-automation for high throughput analysis compared to conventional sample preparation methods often requiring large benchtop systems [15]. Furthermore, as a non-exhaustive extraction technique, it allows for efficient sample clean-up and minimized matrix effects [18].

There is strong interest in discovering predictive biomarkers for PsA vs. PsC using novel ‘omics’ technologies such as metabolomics. Lipids, in particular, are of key interest in psoriatic disease. PsA patients exhibit a higher prevalence of cardiovascular disease, obesity, and dyslipidemia compared to patients with psoriasis alone [5]. Additionally, since previous studies have revealed an abnormal serum fatty acid profile in PsA patients, we sought to develop an SPME-LC-MS method focused on isolating, separating, and analyzing lipids, fatty acids, and fatty acid-like molecules. As a proof of concept, the method was subsequently applied to serum samples from a subset of patients from a larger study who had psoriatic disease. We sought to examine if the method could detect serum lipids and if any significant differences existed between PsC and PsA patients.

## 2. Materials and Methods

LC-MS-grade solvents (acetonitrile, methanol, water, acetonitrile, isopropanol, and acetone), formic acid, concentrated hydrochloric acid, N,N-dimethylformamide (DMF), L-ascorbic acid, dodecanedioic acid, and 12-aminolauric acid were purchased from Thermo Fisher Scientific (New Waltham, MA, USA). The following internal standards and chemicals were purchased from Millipore Sigma (Burlington, MA, USA): amphetamine-d5, MDMA-d5, ketamine-d4, diazepam-d5, oxazepam-d5, codeine-d3, fentanyl-d5, heroin-d9, buprenorphine-d4, nordiazepam-d5, SPLASH LIPIDOMIX Mass Spec Standard, isobutyryl-L-carnitine, and polyacrylonitrile. The standards 1,11-undecanedicarboxylic acid and 10-hydroxy-2-decenoic acid were purchased from Toronto Research Chemicals (Toronto, ON, Canada). Oasis hydrophilic-lipophilic balanced (HLB) particles (50–65 μm) and polystyrene divinylbenzene with weak anion exchanger (PS-DVB-WAX) particles (50–65 μm) were purchased from Waters Corporation (Milford, MA, USA). 

Thin-film stainless steel combs and the Concept-96 manual kit were purchased from PAS technologies (Magdala, Germany). The dip coater and lab oven were purchased from Ni-Lo Scientific (Ottawa, ON, Canada) and Hogentogler (Columbia, SC, USA), respectively. 1 mL deep-well plates were purchased from Canadian Life Science (Peterborough, ON, Canada). A Vanquish autosampler and pump coupled to a Q Exactive Plus Hybrid Quadrupole-Orbitrap Mass Spectrometer, as well as an Accucore C30 HPLC column (100 mm × 2.1 mm, 2.6 μm) and an Accucore C18 HPLC column (50 mm × 2.1 mm, 1.5 μm), were purchased from Thermo Fisher Scientific (New Waltham, MA, USA).

High-performance liquid chromatography with high-resolution mass spectrometry detection was performed using a Vanquish autosampler and pump coupled to a Q Exactive Plus Hybrid Quadrupole-Orbitrap Mass Spectrometer. Chromatographic separation was conducted on an Accucore C30 HPLC column (100 mm × 2.1 mm, 2.6 μm) and an Accucore C18 HPLC column (50 mm × 2.1 mm, 1.5 μm). Thirteen chromatographic methods in positive mode (Table 1) and twelve chromatographic methods in negative mode (Table 2), based on published metabolomic studies focused on lipid metabolism, were tested on both columns.

The Q Exactive Plus Hybrid Quadrupole-Orbitrap Mass Spectrometer included an Ion Max heating source containing a heated electrospray ionization (HESI-II) probe. Mass spectrometer parameters were optimized based on direct infusion with the SPLASH LIPIDOMIX Mass Spec Standard. The mass spectrometer was operated in positive mode at high resolution (70,000), and data was acquired within an *m*/*z* range of 150–1200 with an automatic gain control target of 1 × 10^6^ and an injection time of 100 milliseconds. In negative mode, the mass spectrometer was operated at high resolution (70,000), and data was acquired within an *m*/*z* range of 100–1000 with an automatic gain control target of 1 × 10^6^ and an injection time of 50 milliseconds. The sheath, auxiliary, and sweep gas were set to 48, 11, and 1, respectively, in positive mode and 75, 30, and 1, respectively, in negative mode. The electrospray voltage applied in positive mode was 3.50 kV and −2.60 kV in negative mode. In each method, several lipid standards (Table 3) were assessed for good peak shape (narrow, symmetrical, and gaussian) and good peak resolution. The relative standard deviation (RSD) of the peak area and the retention time were recorded. An injection volume of 5 μL was used for standards; the autosampler temperature was 5 °C.

Serum samples were obtained from the University of Toronto Psoriatic Disease Research Program Biobank. The study received full ethical approval from the University Health Network Research Ethics Board. Patients with PsC (n = 27) and PsA (n = 26) as well as healthy controls (n = 25) were included (see Table 4 for patient demographics). Sample collection took place from 2009 to 2019. Blood was collected in red-top serum separator tubes without additives and allowed to clot at room temperature before centrifugation at 2000× *g* for 15 min. Serum was aliquoted in 0.5 mL vials and frozen at −80 °C until analysis.

The solid-phase microextraction (SPME) device was prepared using a dip-coating method optimized at the Schroeder Arthritis Institute—Centre for Arthritis Diagnostic and Therapeutic Innovation: Metabolomics Core Facility. First, the stainless steel blades were prepared using a previously established protocol [35]. Briefly, the blades were etched in concentrated hydrochloric acid for one hour, rinsed with tap water, and dried overnight in an oven. A 7% w/v polyacrylonitrile (PAN) solution was then prepared in N,N-dimethylformamide (DMF). A specialized software-operated dip-coating machine coated the stainless steel support with a slurry mixture consisting of 7% w/v 1:1 HLB and PS-DVB-WAX particles in PAN solution. The coating was cured in an oven at 150 °C for 1 min after each layer of slurry. The solution was used to coat a total of 32 combs, each of which had 12 blades. Every 8 combs were assembled to create a SPME brush for sample preparation. Each brush was cleaned with 50:25:12.5:12.5 (v/v) water:methanol:acetonitrile:isopropanol and then measured for reproducibility. The final coating on each blade was 2 cm long with an average thickness of 2 mm.

Serum samples were then processed using SPME. Each well of a 96-well plate was filled with 200 μL of serum and 400 μL of phosphate buffered saline (PBS) containing deuterated nordiazepam. The serum samples were then agitated for 30 min at 500 rpm prior to extraction. During this time, the SPME device was placed onto the Concept-96 manual kit and was conditioned in a mixture of 1:1 methanol:water (v/v) for 30 min at 1500 rpm. Subsequent to conditioning, the device underwent a 15 min rinse with water at 1500 rpm, followed by exposure to the serum samples for 1 h at 1500 rpm for extraction. The device underwent another 10 s rinse with water at 500 rpm, and the extracted metabolites were then desorbed for 1 h at 1500 rpm in 600 μL of 4:3:3 methanol:acetonitrile:water (v/v/v) + 0.1% ascorbic acid containing the following deuterated compounds: amphetamine-d5, MDMA-d5, ketamine-d4, diazepam-d5, oxazepam-d5, codeine-d3, fentanyl-d5, heroin-d9, buprenorphine-d4, and SPLASH LIPIDOMIX Mass Spec Standard. The desorption solution was then diluted with 240 μL of water to produce a final extract composed of 1:1 organic/aqueous content (4:3:7 methanol:acetonitrile:water). A total of 10 μL of each sample extract was combined to form a pooled quality control (QC) sample that was injected in the analytical run every 10 sample injections.

Chromatographic separation was conducted on an Accucore C30 HPLC column (100 mm × 2.1 mm, 2.6 μm) using the developed method. Gradient elution in positive mode was accomplished over a 30 min period using mobile phases composed of 99.9 water/0.1 formic acid (v/v) and 99.9 methanol/0.1 formic acid (v/v). Gradient elution in negative mode was accomplished over a 20 min period using mobile phases composed of 99.9 water/0.1 formic acid (v/v) and methanol. During analysis, 5 μL of each sample extract was injected, with the autosampler at 5 °C and the column temperature at 60 °C. Please see Table 5 and Table 6 for additional details on the chromatographic gradient elution. The parameters for mass spectrometry were consistent with those outlined in the assay development. The lipids outlined in Table 3 were prepared in 4:3:7 methanol/acetonitrile/water and used as quality control standards throughout the LC-MS analysis.

The LC-MS data files acquired during instrumental analysis were pre-processed on MetaboAnalyst 5.0. The raw LC-MS data files were first converted to mzML files using MSConvert [36] and then pre-processed using the MetaboAnalyst LC-MS Spectra Processing module [37]. The module performed automated parameter optimization based on pooled quality control (QC) samples run throughout the sequence and supported spectra data processing for peak picking, alignment, and gap filling [37]. The peak lists generated using MetaboAnalyst were initially filtered by removing features with a pooled QC relative standard deviation (RSD) greater than 30%. Further filtering was performed by removing features that had a pooled QC to solvent/device blank ratio of less than 5. The data was uploaded back onto MetaboAnalyst; default settings were used for missing values, with no supplementary filtering applied. A total of 1786 features were detected in positive mode and 1059 features in negative mode. Prior to univariate or multivariate chemometric analyses, a log transformation was performed on the negative mode data, and a cube root transformation in addition to pareto scaling was performed on the positive mode data.

Statistical analysis was performed using MetaboAnalyst 5.0. Since the distribution of the data cannot be assumed, a Wilcoxon Rank test or a Kruskal–Wallis test, both with a false discovery rate (FDR) of 0.05, were applied for univariate analysis. Multivariate analysis included Principal Component Analysis (PCA), Partial Least Squares-Discriminant Analysis (PLS-DA), and Orthogonal Projection to Latent Structures-Discriminant Analysis (OPLS-DA). Model validation for O-PLS-DA and PLS-DA included a combination of permutation (*p* < 0.05 at 2000) and 10-fold cross-validation (10-fold CV). Only features with a variable of importance in projection (VIP) score of >1 were investigated further. Significant features from both univariate and multivariate analyses were tentatively annotated based on exact mass using METLIN [38] and LIPID MAPS [39] in conjunction with the Human Metabolome Database (HMDB) [40]. Global Natural Products Social Molecular Networking (GNPS) [41] and MS-DIAL [42] were used for MS/MS spectral matching. Biochemical importance for all identified compounds was obtained from the Human Metabolome Database (HMDB) [40].

## 3. Results

### 3.1. Method Development

Thirteen chromatographic methods in positive mode and twelve methods in negative mode were tested on both the Accucore C18 HPLC column and the Accucore C30 HPLC column. Overall, both columns provided similar separation of lipids and good peak shapes. The Accucore C30 column resulted in slightly narrower peaks with less tailing. Thus, the Accucore C30 column was selected as the more favorable column for lipid analysis.

Each chromatographic method that was then tested on the Accucore C30 HPLC column had different solvents, additives, and gradients. Many of the methods were not capable of generating a good separation of lipids with a good peak shape. Methods that used water and acetonitrile as mobile phases tended to perform poorly, while methods that used methanol instead of acetonitrile in their mobile phase B were found to generate good separation of lipids and good peak shapes. No singular method, however, was able to capture all lipids adequately. The method that was best for analyzing larger lipids such as glycerophospholipids, glycerolipids, sterol lipids, and sphingolipids was not the best for analyzing smaller lipids such as fatty acids. These differences in lipid analysis may be due to the lipid categories or may be because of the size of the lipids. Method 11 in positive mode and method 8 in negative mode were selected as the chromatographic methods of choice because they provided narrow, symmetrical peaks for most of the fatty acids and fatty acid-like molecules, which were our focus, as mentioned, based on previous research. Regardless, the methods also performed well for many of the other lipids.

The two selected methods were then optimized to achieve the best initial conditions and gradient. The initial percentage of solvent B was increased from 0% to 30%. Both methods showed a similar pattern, where larger initial percentages of B resulted in wider, more asymmetrical peaks. In positive mode, the method showed the best peak shapes at 5% solvent B. In negative mode, solvent B was selected to be the best at 0%. The curvature of each method’s gradient was then modified into a concave down or concave up curve. A concave up gradient increased the retention time of almost all the compounds, while a concave down gradient decreased the retention time. The concave down gradient was selected for both methods because the lower retention time elution of metabolites would allow for more compounds to be separated on the column while preserving good peak shape and a shorter chromatographic method.

**Figure 1:**
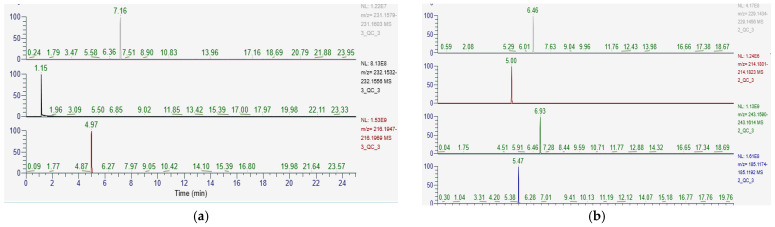
Final optimized methods. (**a**) Positive mode: dodecanedioic acid, isobutyryl-L-carnitine, and 12-aminolauric acid. (**b**) Negative mode: dodecanedioic acid, 12-aminolauric acid, 1,11-undecanedicarboxylic acid, and 10-hydroxy-2-decenoic acid.

The final optimized methods are shown in Figure 1 with the extraction ion chromatograms of our fatty acid-like molecules and derivatives. Although the methods captured many of the lipids with great ability, there were a few lipids that were not observed as narrow, symmetrical peaks (see Table 7 and Table 8 for full list). Dodecanedioic acid and 12-aminolauric acid were well detected in both positive and negative modes. Isobutyryl-L-carnitine was poorly analyzed in negative mode but well analyzed in positive mode. 1,11-undecanedicarboxylic acid and 10-hydroxy-2-decenoic acid were only able to be analyzed in negative mode.

### 3.2. Method Performance

The LC-MS method developed and optimized for separating and analyzing fatty acids and fatty acid-like molecules was subsequently applied to serum samples that were a subset of patients with psoriatic disease from another study and were prepared using SPME. We aimed to examine if the method could detect serum lipids and if there were any differences in serum lipids between PsC and PsA patients. 

Over the course of the analytical run, quality control standards (Table 3) were run in triplicate every ten samples, and the relative standard deviation (RSD) of the peak area and the retention time were recorded. All quality control standards had a peak area RSD < 9% and a retention time RSD < 1%. Deuterated internal standards added to each sample for monitoring metabolite extraction and instrumental analysis had peak area RSDs < 6% and retention time RSDs < 1%. The low variation (<9% and 1% RSD) indicates the method performed well during serum sample instrumental acquisition over a three-day run.

**Figure 2:**
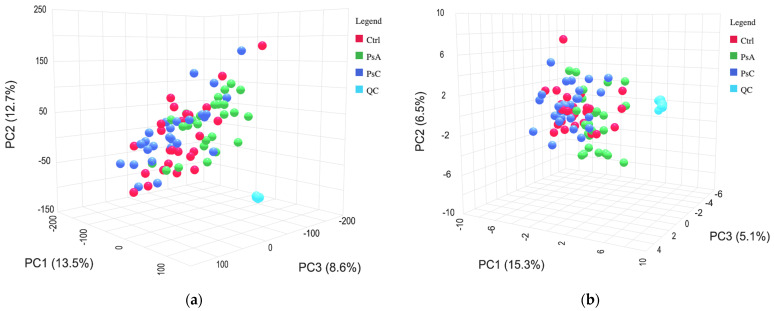
Principal component analysis of pooled QCs, represented by turquoise, and three patient groups—healthy volunteers (Ctrl), patients with psoriatic arthritis (PsA), and patients with psoriasis (PsC), represented by red, green, and dark blue on the plot, respectively. (**a**) Positive mode acquisition. PCA—PC1: 13.5%, PC2: 12.7%, and PC3: 8.6%. (**b**) Negative mode acquisition. PCA—PC1: 15.3%, PC2: 6.5%, and PC3: 5.1%.

Principal component analysis (PCA) was employed to produce a graphical representation of the dataset (Figure 2). PCA is an unsupervised multivariate approach that facilitates the identification of patterns, laying the groundwork for the construction of subsequent supervised models. The pooled QC (a composite of all samples) was injected throughout the analysis every 10 samples. The data indicated tight clustering of the QCs on the PCA plot of both positive and negative mode data, which indicates instrumental stability during acquisition.

### 3.3. Significant Differences between Patient Groups

**Figure 3:**
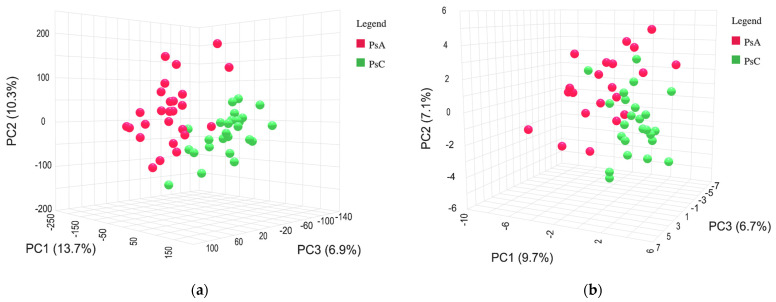
Principal component analysis of patients with psoriatic arthritis (PsA) and patients with psoriasis (PsC), represented by red and green on the plot, respectively. (**a**) Positive mode acquisition. PCA—PC1: 13.7%, PC2: 10.3%, and PC3: 6.9%. (**b**) Negative mode acquisition. PCA—PC1: 9.7%, PC2: 7.1%, and PC3: 6.7%.

**Figure 4:**
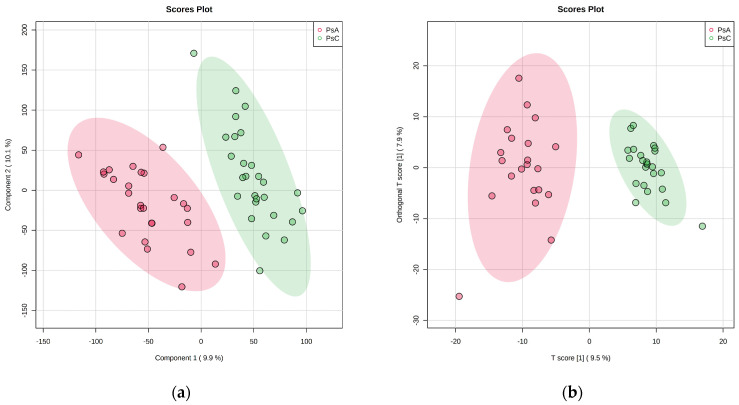
Discriminant analysis of patients with psoriatic arthritis (PsA) and patients with psoriasis (PsC), represented by red and green on the plot, respectively. (**a**) Partial least squares discriminant analysis (PLS-DA) of positive mode acquisition data. The model fits the acceptable criteria of 0.89 (R^2^) and 0.77 (Q^2^). (**b**) Orthogonal projections to latent structures discriminant analysis (OPLS-DA) of negative mode acquisition data. The model fits the acceptable criteria of 0.77 (R^2^) and 0.61 (Q^2^).

The PCA plots show a clear separation between the PsA group and the PsC group in both positive and negative modes (Figure 3). Multivariate analysis of PsA and PsC patients via PLS-DA yielded a cross-validated model (Figure 4) with acceptable criteria of 0.89 (R^2^) and 0.77 (Q^2^) in positive mode. In negative mode, multivariate analysis of PsA and PsC patients via OPLS-DA yielded a cross-validated model with acceptable criteria of 0.77 (R^2^) and 0.61 (Q^2^) (Figure 4). Features with a variable of importance in projection (VIP) score of >1 had the most significant influence on each model. Statistical analysis via univariate analysis yielded sixteen statistically significant features in positive mode and nine statistically significant features in negative mode between PsC and PsA patients. Features that were significant via univariate analysis and had a VIP score of >1 in the multivariate discriminant analysis were identified. See Table 9 and Table 10 for a complete list of confirmed and tentatively identified compounds, respectively, in positive mode. See Table 11 for a complete list of tentatively identified compounds in negative mode.

## 4. Discussion

The aim of this study was to develop an SPME-LC-MS untargeted metabolomic method focused on isolating, separating, and analyzing lipids—with a specific focus on fatty acids and fatty acid-like molecules. These compounds were selected because they have previously been found to be implicated in psoriatic disease and may aid in classifying PsA patients from PsC patients [6,10]. Additionally, the high prevalence of metabolic syndrome and cardiovascular events in PsA patients makes lipids of key interest in psoriatic disease. SPME-LC-MS was chosen as the analytical assay due to the streamlined, semi-automated workflow, the varying compound classes capable of being extracted, and previous research that has shown SPME to be comparable to common lipid extraction methods such as Folch extraction and Bligh and Dyer [43].

Thirteen chromatographic methods in positive mode and twelve methods in negative mode, with varying solvents, additives, and gradients, were tested on two HPLC columns. Each method’s performance was evaluated using instrumental quality control standards. The Accucore C30 column provided slightly narrower peaks and was thus chosen as the most suitable column for analysis. Chromatographic methods that used methanol instead of acetonitrile in their mobile phase B performed better. No method, however, was able to capture all lipids perfectly. The method chosen in each acquisition mode was selected because it was best able to separate and analyze fatty acid-like molecules, which are a priority for further investigation in psoriatic disease. The initial conditions for solvent B were then optimized and achieved the narrowest peaks at smaller initial percentages. A concave downward gradient was selected because it reduced retention times while preserving peak shape.

After developing and optimizing the final method in positive and negative modes, the methods were applied to serum samples collected from a cohort of patients with PsC, PsA, and healthy controls. The analytical run was evaluated using instrumental quality control standards, deuterated internal standards, and pooled quality control samples. A low variation in the RSD of the peak and retention time of the standards indicated a stable run. We examined significant metabolite differences between PsC and PsA patients to help understand how PsA patients may be able to be better detected among patients with psoriatic disease. Tentatively annotated features classified as fatty acids, carboxylic acids, glycerophospholipids, steroids, and sphingolipids were statistically significant between the PsC and PsA groups using both multivariate and univariate approaches. 

The short-chain acylcarnitine, valerylcarnitine, was the only compound that had its identity confirmed using MS/MS spectra (See Figure S1, S2 and S3). Another acylcarnitine, acetyl-D-carnitine, was found to differentiate PsA and PsC patients; however, this tentative feature was not able to be confirmed using MS/MS. Acylcarnitines are involved in transporting fatty acids into the mitochondria to be broken down to produce energy via beta-oxidation. Previous studies have found changes in the levels of acylcarnitines in patients with diabetes [44,45], obesity [45], and heart disease [46], which are all comorbidities of psoriatic disease. Interestingly, a recent study by Villarreal-Martinez revealed that PsC patients with insulin resistance had a distinct acylcarnitine profile from patients without insulin resistance, reflecting impaired beta-oxidation [47]. It appears acylcarnitines may be a possible connection between dysregulated fatty acid metabolism seen in previous metabolomic studies and the high prevalence of metabolic abnormalities in patients with psoriatic disease.

Other tentatively identified features included tryptophyl-histidine, isoleucyl-isoleucine, phenylalanyl-tryptophan, and arginyl-histidine. These four compounds are dipeptides, formed as a result of an incomplete breakdown in protein catabolism. No previous studies have been published regarding these compounds. Additionally, we found that levels of both lysophosphatidic acid (18:2(9Z,12Z)/0:0) and sphingomyelin (d18:1/24:0) distinguished PsA and PsC patients. Lysophosphatidic acid (LPA) is an important intercellular lipid mediator controlling wound healing and the inflammatory cascade [48]. A previous study revealed LPA levels at significantly higher levels in psoriatic patients’ serum and suggested LPA may mediate the pathogenesis of psoriasis by activating keratinocytes [49]. The role of LPA in PsA has not yet been reported in the literature; however, our study points to a possible implication in the disease. Sphingomyelin (SM) is a sphingolipid and a component of animal cell membranes [50]. A previous metabolomic study detected an increase in serum concentrations of sphingolipids in PsC patients with severe disease activity [51].

It is important to recognize, however, that this study and untargeted metabolomic studies in general are not without their limitations. Identification of features remains a roadblock in untargeted metabolomics. In this study, we employed several databases for feature identification; however, we were only able to tentatively identify approximately 20% of significant features. Out of the 18 tentatively identified features, we were able to confirm the identity of one compound using MS/MS spectra. This could be due to a few reasons, including the dearth of MS/MS fragmentation databases as well as the sheer difficulty of identifying a very diverse and complex set of molecules like lipids. Although we developed the SPME-LC-MS method for analyzing lipids and lipid-related molecules, without a targeted approach, any metabolite with the appropriate physical and chemical properties may be detected, including those with exposome-borne origins. One of the great strengths of metabolomics for biomarker discovery is that it considers genetic, immunologic, and environmental factors. However, given the complexity of the metabolome, it can be difficult to determine which factors contribute largely to the disease’s manifestation. A multitude of intrinsic and extrinsic factors, such as diet, ethnicity, exercise, past drug use, and hormonal status, can contribute to a patient’s metabolome. In this study, we included three patient groups of approximately the same sample size: sex-balanced, age, and BMI-adjusted; however, we did not control these additional factors due to limited sample availability. 

## 5. Conclusions

The SPME-LC-MS method developed and optimized was capable of detecting lipids, fatty acids, fatty acid-like molecules, and other compounds that may aid in differentiating psoriasis and PsA patients. Several classes of lipids detected (acylcarnitines, lysophospholipids, and sphingolipids) have previously been indicated in psoriatic disease. Specific focus should be placed on valerylcarnitine due to its confirmed identity. This preliminary data provides a good direction for future research in lipid biomarker discovery in psoriatic disease. Due to this study’s relatively small sample size, a more expansive follow-up study should be conducted to confirm the findings. This expanded study should use the same SPME-LC-MS method but increase the number of patients examined and include rigorous MS/MS validation.

## Figures and Tables

**Table 1:** Liquid chromatographic gradients used for separation in positive mode.

Method	Study	Mobile Phase A	Mobile Phase B	Gradient Length (min)	Flow Rate (mL/min)	Column Temp. (°C)
1	[21,22]	60% ACN 40% H_2_O + 10 mM ammonium formate + 0.1% formic acid	90% IPA 10% ACN + 10 mM ammonium formate + 0.1% formic acid	31	0.3	50
2	N/A	60% ACN 40% H_2_O + 10 mM ammonium formate	90% IPA 10% ACN + 10 mM ammonium formate	31	0.3	50
3	[23]	60% H_2_O 40% MeOH + 10 mM ammonium acetate + 1 mM acetic acid	90% IPA 10% MeOH + 10 mM ammonium acetate + 1 mM acetic acid	50	0.2	55
4	N/A	60% H_2_O 40% MeOH + 10 mM ammonium formate + 1 mM formic acid	90% IPA 10% MeOH + 10 mM ammonium formate + 1 mM formic acid	50	0.2	55
5	N/A	60% H_2_O 40% MeOH + 10 mM ammonium formate	90% IPA 10% MeOH + 10 mM ammonium formate	50	0.2	55
6	[24]	50% ACN 50% H_2_O + 5 mM ammonium formate	85% IPA 10% ACN 5% H_2_O + 5 mM ammonium formate	32	0.325	50
7	[25]	H_2_O + 0.1% formic acid	ACN	45	0.2	30
8	[26]	H_2_O + 0.1% formic acid	ACN + 0.1% formic acid	30	0.5	50
9	[27]	H_2_O + 0.1% formic acid	ACN + 0.1% formic acid	57	0.05	50
10	[28]	H_2_O + 0.1% formic acid	ACN + 0.1% formic acid	22	0.4	40
11	[29]	H_2_O + 0.1% formic acid	MeOH + 0.1% formic acid	25	0.4	60
12	[30]	H_2_O + 12 mM ammonium acetate + 0.02% acetic acid	90% ACN 10% H_2_O + 12 mM ammonium acetate + 0.02% acetic acid	35	0.5	30
13	[31]	H_2_O + 20 mM ammonium formate	60% IPA 36% ACN 4% H_2_O + 0.1% formic acid	25	0.4	60

Abbreviations: ACN—acetonitrile; H_2_O—water; IPA—isopropanol; MeOH—methanol; Temp.—temperature.

**Table 2:** Liquid chromatographic gradients used for separation in negative mode.

Method	Study	Mobile Phase A	Mobile Phase B	Gradient Length (min)	Flow Rate (mL/min)	Column Temp. (°C)
1	[21,22]	60% ACN 40% H_2_O + 10 mM ammonium formate + 0.1% formic acid	90% IPA 10% ACN + 10 mM ammonium formate + 0.1% formic acid	31	0.3	50
2	N/A	60% ACN 40% H_2_O + 10 mM ammonium formate	90% IPA 10% ACN + 10 mM ammonium formate	31	0.3	50
3	[23]	60% H_2_O 40% MeOH + 0.02% acetic acid	90% IPA 10% MeOH + 0.02% acetic acid	50	0.2	55
4	[24]	50% ACN 50% H_2_O + 5 mM ammonium formate	85% IPA 10% ACN 5% H_2_O + 5 mM ammonium formate	32	0.325	50
5	[27]	H_2_O + 0.1% formic acid	ACN + 0.1% formic acid	57	0.05	50
6	[28]	H_2_O + 0.1% formic acid	ACN + 0.1% formic acid	22	0.4	40
7	[29]	H_2_O + 0.1% formic acid	MeOH + 0.1% formic acid	25	0.4	60
8	[32]	H_2_O + 0.1% formic acid	MeOH	15	0.4	50
9	[30]	H_2_O + 12 mM ammonium acetate + 0.02% acetic acid	90% ACN 10% H_2_O + 12 mM ammonium acetate + 0.02% acetic acid	35	0.5	30
10	[31]	H_2_O + 20 mM ammonium formate	60% IPA 36% ACN 4% H_2_O + 0.1% formic acid	25	0.4	60
11	[33]	60% ACN 40% H_2_O + 10 mM ammonium acetate	50% ACN 50% IPA + 10 mM ammonium	23	0.450	55
12	[34]	90% H_2_O 10% MeOH	80% MeOH 20% ACN	8	0.3	50
13	[21,22]	60% ACN 40% H_2_O + 10 mM ammonium formate + 0.1% formic acid	90% IPA 10% ACN + 10 mM ammonium formate + 0.1% formic acid	31	0.3	50

Abbreviations: ACN—acetonitrile; H_2_O—water; IPA—isopropanol; MeOH—methanol; Temp.—temperature.

**Table 3:** Standards analyzed in positive and negative modes.

Compound	Lipid Category	*m*/*z* Positive Mode	*m*/*z* Negative Mode
15:0-18:1(d7) PC	Glycerophospholipids	753.6134	N/A
18:1(d7) Lyso PC	Glycerophospholipids	529.3994	N/A
15:0-18:1(d7) PE	Glycerophospholipids	711.5664	709.5519
18:1(d7) Lyso PE	Glycerophospholipids	487.3524	485.3379
15:0-18:1(d7) PG	Glycerophospholipids	759.5875	740.5464
15:0-18:1(d7) PI	Glycerophospholipids	847.6036	828.5625
15:0-18:1(d7) PS	Glycerophospholipids	755.5562	753.5417
15:0-18:1(d7)-15:0 TAG	Glycerolipids	829.7985	N/A
15:0-18:1(d7) DAG	Glycerolipids	605.5844	N/A
18:1(d7) MAG	Glycerolipids	364.3429	N/A
18:1(d7) Chol Ester	Sterol Lipids	675.6779	N/A
d18:1-18:1(d9) SM	Sphingolipids	738.647	N/A
15:0-18:1(d7) PA	Glycerophospholipids	N/A	666.5097
Cholesterol-d7	Sterol Lipids	411.4326	N/A
Dodecanedioic acid	Fatty Acyls	231.1591	229.1445
Isobutyryl-L-carnitine	Fatty Acyls	232.1544	230.1398
12-Aminolauric acid	Fatty Acyls	216.1958	214.1812
1,11-Undecanedicarboxylic acid	Fatty Acyls	N/A	243.1602
10-Hydroxy-2-decenoic acid	Fatty Acyls	N/A	185.1183

**Table 4:** Patient demographics.

Characteristic	Healthy Controls	Psoriasis	Psoriatic Arthritis
Number of patients	25	27	26
Number of females (%)	40	51	46
Mean age (years)	48	43	47
BMI	29	27	26

**Table 5:** Liquid chromatographic gradient used for separation in positive mode.

Time (min)	% Mobile Phase B (Methanol + 0.1% Formic Acid)
0	5
1	5
20	100 (curve 3)
22.5	100
25	5
30	5

**Table 6:** Liquid chromatographic gradient used for separation in negative mode.

Time (min)	% Mobile Phase B (Methanol + 0.1% Formic Acid)
0	0
2	0
12	100 (curve 3)
15	100
18	0
20	0

**Table 7:** Standards analyzed in the final method—positive mode.

Compound	Adduct	*m*/*z*	RT (min)
Dodecanedioic acid	[M + H]^+^	231.1591	7.16
Isobutyryl-L-carnitine	[M + H]^+^	232.1544	1.15
12-Aminolauric acid	[M + H]^+^	216.1958	4.97

**Table 8:** Standards analyzed in the final method—negative mode.

Compound	Adduct	*m*/*z*	RT (min)
Dodecanedioic acid	[M − H]^−^	229.1445	6.46
12-Aminolauric acid	[M − H]^−^	214.1812	5.00
1,11-Undecanedicarboxylic acid	[M − H]^−^	243.1602	6.93
10-Hydroxy-2-decenoic acid	[M − H]^−^	185.1183	5.47

**Table 9:** Confirmed compounds using MS/MS showing statistically significant differences between psoriasis and psoriatic arthritis patients during positive mode acquisition.

m/z	RT (min)	Confirmed ID	Adduct	Biochemical Importance
246.1699	3.2	Valerylcarnitine	[M+H]^+^	Short-chain acylcarnitine involved in beta-oxidation.

**Table 10:** Tentatively annotated features showing statistically significant differences between psoriasis and psoriatic arthritis patients during positive mode acquisition.

m/z	RT (min)	Tentative ID	Adduct	Biochemical Importance
342.1558	1.5	Tryptophyl-Histidine	[M+H]^+^	Dipeptide resulting from an incomplete breakdown in protein catabolism.
457.2308	9.1	LPA(18:2(9Z,12Z)/0:0)	[M+Na]^+^	Intercellular lipid mediator.
473.1933	4.2	Sofalcone	[M+Na]^+^	Mucosal protective agent.
491.2125	4.2	Cymorcin diglucoside	[M+H]^+^	Phenolic glycoside.
489.2246	3.5	Dolichyl b-D-glucosyl phosphate	[M+Na]^+^	Sesquiterpenoid.
374.147	3.8	Phenylalanyl-Tryptophan	[M+Na]^+^	Dipeptide resulting from an incomplete breakdown in protein catabolism.
361.2007	5.1	Aldosterone	[M+H]^+^	Aldosterone is a hormone that regulates sodium and potassium.
334.1588	14.3	Arginyl-Histidine	[M+Na]^+^	Dipeptide resulting from an incomplete breakdown in protein catabolism.
780.5307	7.6	(3′-sulfo)Galbeta-Cer(d18:1/16:0)	[M+H]^+^	Found in particularly high concentrations in myelin.
815.6991	22.1	SM(d18:1/24:0)	[M+H]^+^	Sphingolipids were found in cell membranes. Role in signal transduction.
204.123	0.9	Acetyl-D-carnitine	[M+H]^+^	Long-chain acylcarnitine involved in beta-oxidation.
391.2089	4.8	19R-hydroxy-PGE2, 20-hydroxy-PGE2	[M+Na]^+^	PGE2 stimulates bone resorption by osteoclasts, increases vasodilation, and increases cAMP production.

**Table 11:** Tentatively annotated features showing statistically significant differences between psoriasis and psoriatic arthritis patients during negative mode acquisition.

m/z	RT (min)	Tentative ID	Adduct	Biochemical Importance
243.1715	4.1	Isoleucyl-Isoleucine	[M-H]^-^	Dipeptide resulting from an incomplete breakdown in protein catabolism.
935.3194	0.8	Bilirubin diglucuronide	[M-H]^-^	Glucuronidated derivative of bilirubin.
997.3346	0.8	Sialyllacto-N-tetraose a	[M-H]^-^	Oligosaccharide found in breast milk.
350.1517	4.4	Tryptophyl-Phenylalanine	[M-H]^-^	Dipeptide resulting from an incomplete breakdown in protein catabolism.
179.0565	0.9	2,3-Dihydroxy-2-methylbutanoic acid	[M+Formate]^-^	Hydroxy fatty acid.

## Data Availability

The data have been deposited in the EMBL-EBI MetaboLights database (DOI: 10.1093/nar/gkz1019, PMID: 31691833) with the identifiers MTBLS6871 and MTBLS6901 and is currently under review. In the meantime, all processed data will be available on request from the corresponding author.

## Supplementary Materials

The following supporting information can be downloaded at: https://www.mdpi.com/article/10.3390/metabo13080963/s1, Figure S1: extracted ion chromatogram for valerylcarnitine; Figure S2: measured versus reference MS/MS spectra for valerylcarnitine. Figure S3: proposed fragmentation pattern for valerylcarnitine.
