# Supplementary material for: A Solid-Phase Microextraction—Liquid Chromatography-Mass Spectrometry Method for Analyzing Serum Lipids in Psoriatic Disease
Source: Metabolites. 2023 Aug 20;13(8):963. doi: 10.3390/metabo13080963 (PMC10456752; doi:10.3390/metabo13080963)
Supplement: Supplementary file 1 [file metabolites-13-00963-s001.zip › metabolites-2531500-supplementary.pdf]

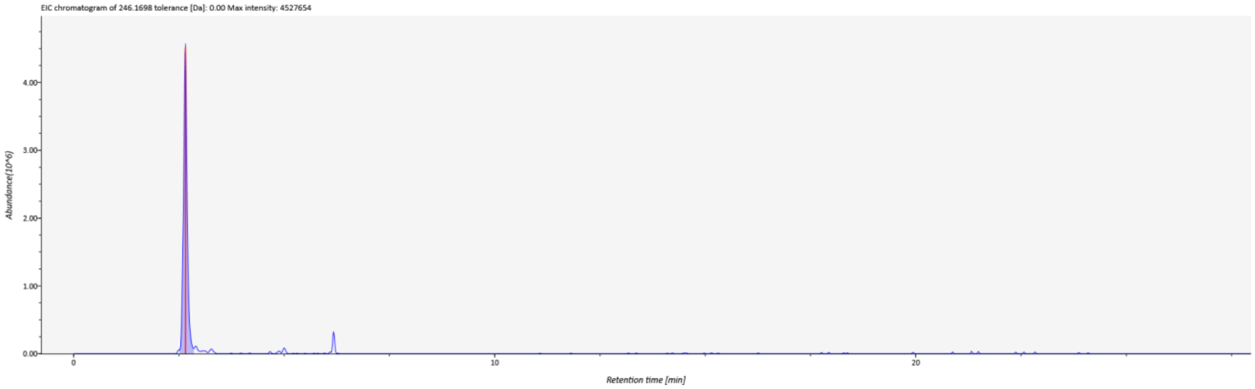

Figure S1. Extracted ion chromatogram for valerylcarnitine.

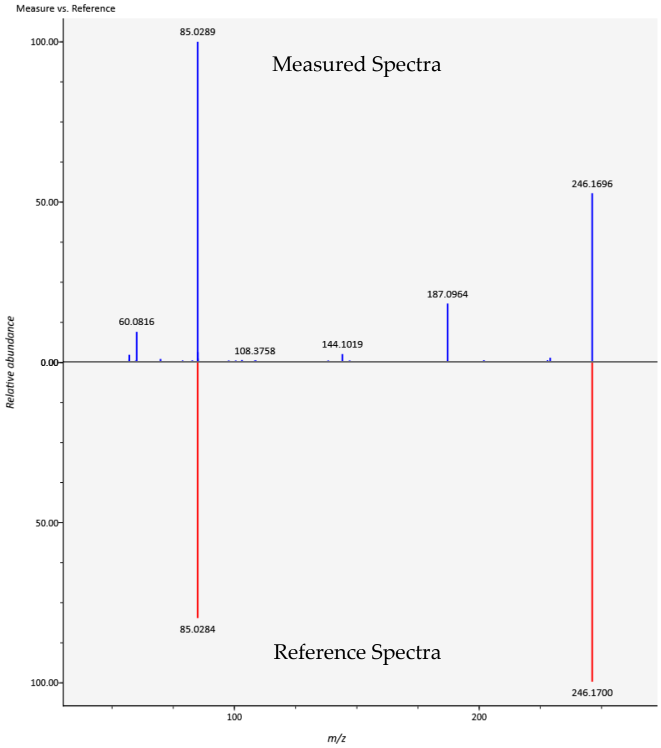

Figure S2. Measured versus reference MS/MS spectra for valerylcarnitine.

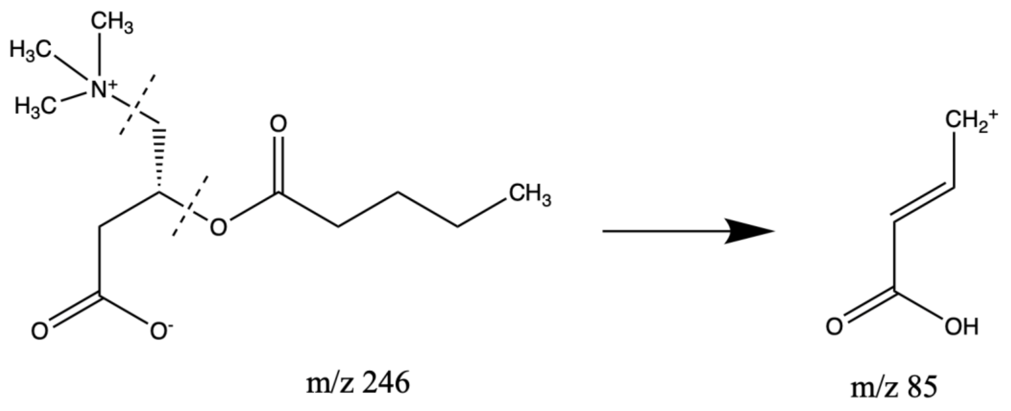

Figure S3. Proposed fragmentation pattern for valerylcarnitine.
